# COVID-19 in Kidney Transplant Recipients: A Multicenter Experience from the First Two Waves of Pandemic

**DOI:** 10.1186/s12882-022-02784-w

**Published:** 2022-05-12

**Authors:** Erol Demir, Zuhal Atan Ucar, Hamad Dheir, Ramazan Danis, Berna Yelken, Murathan Uyar, Ergun Parmaksiz, Ayse Serra Artan, Ayse Sinangil, Ozgur Merhametsiz, Serap Yadigar, Ahmet Burak Dirim, Baris Akin, Nurana Garayeva, Seda Safak, Aydin Turkmen

**Affiliations:** 1 Department of Internal Medicine, Division of Nephrology, Istanbul Faculty of Medicine, Istanbul University, Istanbul, Turkey; 2 Department of Internal Medicine, Division of Nephrology, Florence Nightingale Hospital, Bilim University, Istanbul, Turkey; 3 Department of Internal Medicine, Division of Nephrology, Sakarya University Training and Research Hospital, Sakarya, Turkey; 4 Department of Internal Medicine, Division of Nephrology, Diyarbakir Gazi Yasargil Training and Research Hospital, Diyarbakir, Turkey; 5 Department of Internal Medicine, Division of Nephrology, Koc University School of Medicine, Istanbul, Turkey; 6 Department of Internal Medicine, Division of Nephrology, T.C. Istanbul Yeni Yuzyil University, Gaziosmanpasa Hospital, Istanbul, Turkey; 7 Department of Internal Medicine, Division of Nephrology, Kartal Dr. Lutfi Kirdar Training and Research Hospital, Istanbul, Turkey

**Keywords:** COVID-19, Kidney transplantation, Anti-viral agents, Cytokine-targeted therapy, SARS-CoV-2, Acute respiratory distress syndrome

## Abstract

**Background:**

Kidney transplant recipients have an increased risk of complications from COVID-19. However, data on the risk of allograft damage or death in kidney transplant recipients recovering from COVID-19 is limited. In addition, the first and second waves of the pandemic occurred at different times all over the world. In Turkey, the Health Minister confirmed the first case in March 2020; after that, the first wave occurred between March and August 2020; afterward, the second wave began in September 2020. This study aims to demonstrate the clinical presentations of kidney transplant recipients in the first two waves of the pandemic in Turkey and explore the impact of COVID-19 on clinical outcomes after the initial episode.

**Methods:**

Patients with COVID-19 from seven centers were included in this retrospective cohort study. Initially, four hundred and eighty-eight kidney transplant recipients diagnosed with COVID-19 between 1 March 2020 to 28 February 2021 were enrolled. The endpoints were the occurrence of all-cause mortality, acute kidney injury, cytokine storm, and acute respiratory distress syndrome. In addition, longer-term outcomes such as mortality, need for dialysis, and allograft function of the surviving patients was analyzed.

**Results:**

Four hundred seventy-five patients were followed up for a median of 132 days after COVID-19. Forty-seven patients (9.9%) died after a median length of hospitalization of 15 days. Although the mortality rate (10.1% vs. 9.8%) and intensive care unit admission (14.5% vs. 14.5%) were similar in the first two waves, hospitalization (68.8% vs. 29.7%; *p* < 0.001), acute kidney injury (44.2% vs. 31.8%; *p* = 0.009), acute respiratory distress syndrome (18.8% vs. 16%; *p *= 0.456), and cytokine storm rate (15.9% vs. 10.1%; *p* = 0.072) were higher in first wave compared to the second wave. These 47 patients died within the first month of COVID-19. Six (1.4%) of the surviving patients lost allografts during treatment. There was no difference in the median serum creatinine clearance of the surviving patients at baseline (52 mL/min [IQR, 47–66]), first- (56 mL/min [IQR, 51–68]), third- (51 mL/min [IQR,48–67]) and sixth-months (52 mL/min [IQR, 48–81]). Development of cytokine storm and posttransplant diabetes mellitus were independent predictors for mortality.

**Conclusions:**

Mortality remains a problem in COVID-19. All the deaths occur in the first month of COVID-19. Also, acute kidney injury is common in hospitalized patients, and some of the patients suffer from graft loss after the initial episode.

## Introduction

Severe acute respiratory syndrome coronavirus 2 (SARS-CoV-2) that caused coronavirus disease 2019 (COVID‐19) was detected in China in December 2019, then triggered the pandemic in March 2020 [1]. In addition, the first and second waves of the pandemic occurred at different times all over the world. In Turkey, the Health Minister confirmed the first case in March 2020; after that, the first wave occurred between March and August 2020; afterward, the second wave began in September 2020. According to data from the Health Ministry of Turkey, about 3 million patients were infected, 30,000 patients died from March 2020 to March 2021 [2]. Major risk factors for death were older age, obesity, diabetes, and malignancy [3]. Lymphopenia, acute kidney injury, and high inflammatory markers in COVID-19 are associated with worse outcomes. The clinical presentation of COVID-19 is variable and ranges from asymptomatic infection to pneumonia, cytokine storm, and death [4]. Diagnosis is based on clinical and laboratory findings. Radiological and molecular techniques are used to confirm the diagnosis [4]. Dexamethasone, tocilizumab, and baricitinib have been shown to reduce mortality in the treatment of COVID-19, and remdesivir shortens the length of hospital stay [5]. Also, treatment recommendations have been revised over time based on the latest clinical trials results.

Kidney transplant recipients have a high risk of complications from COVID-19 because of immunosuppression and comorbid diseases [6, 7]. However, matched control studies have disputed the contributory role of immunosuppression for outcomes [6, 8]. The clinical spectrum of COVID-19 in kidney transplant recipients may differ depending on the previous induction and anti-rejection therapies, maintenance immunosuppression, and recipient comorbidities [6]. Delays in diagnosis may occur due to disregarding slight symptoms and misleading laboratory abnormalities. An interdisciplinary team that can optimize the immunosuppression, treat the infection, and gives supportive care should provide COVID-19 treatment [6, 8]. However, an optimal immunosuppression regimen has not been defined yet. In addition, there is not enough information about the differences in the first and second periods of the pandemic in kidney transplant recipients (9, 10). In addition, data on the risk of allograft damage or death in kidney transplant recipients recovering from COVID-19 are limited. This multicenter cohort study aims to present the clinical presentations of kidney transplant recipients in the first two waves of the pandemic in Turkey, identify predictors of worse outcomes, investigate the effect of COVID-19 on clinical outcomes after the initial acute episode.

## Material and method

### Study population

**Fig. 1:**
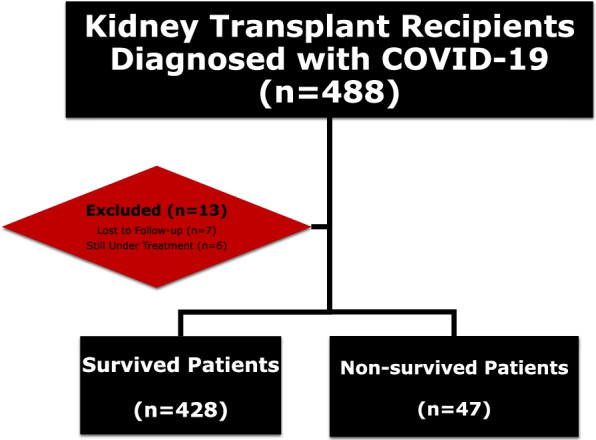
Flowchart of the patients in the study. Abbreviations: COVID-19; Coronavirus disease 2019

**Fig. 2:**
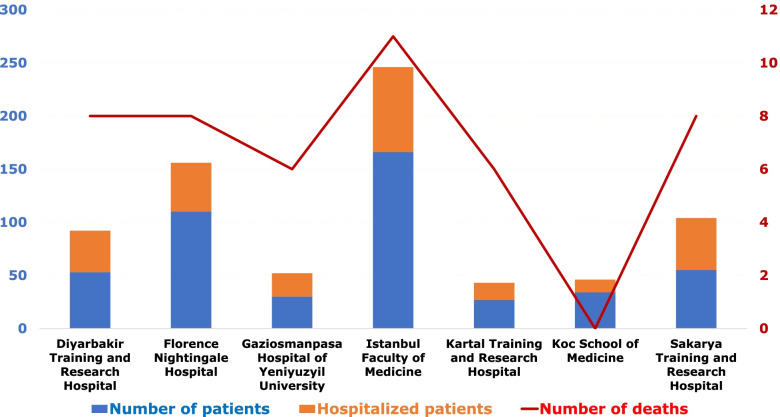
The distribution of the COVID-19 patients according to transplant centers

This retrospective cohort study included kidney transplant recipients with COVID-19 from seven centers in Turkey. Initially, four hundred and eighty-eight kidney transplant recipients diagnosed with COVID-19 between 1 March 2020 to 28 February 2021 were enrolled. The exclusion criteria included patients who were lost to follow-up after diagnosis (*n *= 7) or still under treatment due to COVID-19 (*n* = 6). Four hundred and seventy-five participants were followed up for at least 28 days or until death, as shown in Fig. 1. Figure 2 shows the distribution of the patients according to their transplant centers. The Medical Ethics Committee of the Istanbul Faculty of Medicine approved this study.

### Data collection

Demographic data (age, sex, comorbidities, etiology of primary kidney disease, duration of post-transplant follow-up, type of donor, induction therapy, maintenance immunosuppression, and medication), clinical characteristics (duration of hospitalization, presenting symptoms, contact history, symptoms, examination findings, and laboratory results), and medication during COVID-19 (antiviral, antibiotic, cytokine‐targeted, anticoagulation treatment, and oxygen therapy) were extracted from electronic medical records. Data from out and hospitalized patients were collected. Patients admitted to the hospital excluded those requiring intensive care unit admission.

### Patient management

The patients were diagnosed with COVID-19 based on clinical and laboratory findings. Positive results on real-time polymerase chain reaction (RT-PCR) assay of nasopharyngeal swab specimens or compatible findings on a computed chest tomography scan were used to confirm the diagnosis. In the first three months of the pandemic, patients with a high clinical suspicion of disease were diagnosed by computed tomography of the chest due to low RT-PCR sensitivity [9].

All patients underwent blood testing at admission. Laboratory investigations included a complete blood count and serum C-reactive protein, interleukin-6, markers of myocardial damage (creatine kinase, troponin I, lactate dehydrogenase), tests of secondary hemostasis profile (prothrombin time, activated partial thromboplastin time), serum biochemical tests (including renal and liver function, and electrolytes), procalcitonin, fibrinogen, d-dimer. Laboratory tests were repeated daily for inpatients and weekly for outpatients for two weeks. The estimated glomerular filtration rate (eGFR) was calculated by the modification of diet in the renal disease study equation (MDRD).

Patients were divided into four groups according to the severity of the disease per our national guideline [10]. Asymptomatic patients and patients with mild disease (oxygen saturation above 93%, no lung involvement on chest computed tomography) were followed up in the outpatient clinic weekly. Patients with moderate disease (oxygen saturation above 90%, respiratory rate under 30 breaths/min) were hospitalized in the first wave of the pandemic and followed up in the outpatient clinic in the later period because of hospital overcrowding.

Also, indications for hospitalization were severe disease (oxygen saturation under 90%, respiratory rate above 30 breaths/min), cytokine storm (persistent fever, high or increasing CRP, ferritin, and D-dimer, abnormalities in liver function tests, hypofibrinogenemia with cytopenia in the forms of lymphopenia and thrombocytopenia). Criteria for admission to the intensive care unit (ICU) were the low partial pressure of arterial oxygen, the inspiratory oxygen fraction (PaO_2_ / FiO_2_) ratio less than 300, oxygen saturation less than 90%, and PaO_2_ below 70 mm Hg despite 5 L/minute oxygen therapy, and persistent hypotension (systolic blood pressure < 90 mm Hg or mean arterial pressure < 65 mm Hg).

The cytokine storm was diagnosed according to our national guideline. In this way, cytokine‐targeted therapy indications were standardized for all centers, and problems in drug supply were avoided [10]. Acute kidney injury was classified according to the Kidney Disease Improving Global Outcomes (KDIGO) guideline [11].

### Treatment regimens

**Fig. 3:**
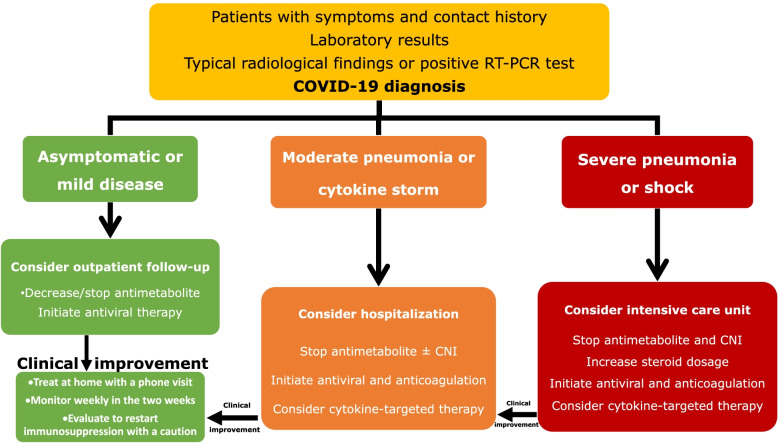
Treatment scheme for kidney transplant recipients with COVID-19. Abbreviations: COVID-19; Coronavirus disease 2019, RT-PCR; Reverse transcription-polymerase chain reaction, CNI; Calcineurin inhibitor

A protocol was used to manage immunosuppression, antiviral, and cytokine‐targeted therapy, shown in Fig. 3. If the patient is asymptomatic, the immunosuppressive regimen does not change. Antimetabolites (mycophenolate derivatives and azathioprine) were discontinued in symptomatic cases. Trough levels were adjusted as 4–6 ng/dL for tacrolimus, 25–75 ng/dL for cyclosporine, and 3–7 ng/dL for everolimus for a patient with stable clinic course and were stopped in hypoxemic patients. The calcineurin inhibitor and mammalian target of rapamycin inhibitor levels were also monitored twice a week in hospitalized patients, once a week in outpatients.

The patients were treated with hydroxychloroquine (400 mg twice a day for the first, and then 200 mg twice a day for four days via oral administration) and azithromycin (500 mg once a day for the first, and then 250 mg once a day for four days via oral administration) in the first wave of the pandemic. Then, favipiravir (1600 mg twice a day for the first, and then 600 mg twice a day for four days via oral administration) was used during the second wave of the pandemic.

Tocilizumab (400–800 mg once a day for two days via intravenous infusion) or anakinra (100–600 mg once a day for seven to fourteen days or until hospital discharge via subcutaneous injection) was used for cytokine storm treatment. Convalescent plasma and intravenous immunoglobulin (IVIG) were used in the absence of anakinra or tocilizumab. Antibiotic therapy was administered based on the infection specialist’s decision in the presence of confirmed or suspected invasive bacterial infection. The patients were monitored for adverse drug reactions during the hospital stay. Also, QT interval measurements were obtained in all patients by the 12-lead electrocardiography.

### Supportive care

Prophylactic-dose low-molecular-weight heparin was used for inpatients unless there were contraindications. Patients with deep vein thrombosis or pulmonary embolism were treated with therapeutic-dose anticoagulation. Doses were adjusted according to the bleeding risk of the patient.

Oxygen treatment was provided to patients whose oxygen saturation was below 92% via a nasal cannula and with a non-rebreather mask if the former was insufficient. If respiratory failure persisted despite these treatments, invasive mechanical ventilation would be used after non-invasive ventilation. All hypoxemic patients were treated with steroids (40 mg methylprednisolone once a day via intravenous infusion in the first wave of the pandemic and 6 mg dexamethasone once a day intravenously after the RECOVERY trial) [12].

### Follow-up protocol after discharge

Hospitalized patients were discharged after resolving hypoxia and cytokine storm. Complete blood count and serum C-reactive protein, serum biochemical tests (including renal and liver function, and electrolytes) were monitored weekly after discharge. The calcineurin inhibitor was added to the patients in good condition during the first-week clinical visit. Antimetabolites were restarted at week two in patients with a stable clinical course, and doses were gradually increased at subsequent clinical visits. Trough levels were set at 5–10 ng/dL for tacrolimus and 50–150 ng/dL for cyclosporine in discharged patients.

### Outcomes

The primary endpoint was all-cause mortality. The secondary endpoints were acute kidney injury, cytokine storm, and acute respiratory distress syndrome. All endpoints were classified according to the first and second waves. In addition, longer-term outcomes such as mortality, need for dialysis, and allograft function of the patients who recovered and were discharged was analyzed.

### Statistical analysis

Patients were categorized as survivors and non-survivors. All parameters such as patient demographic, clinical presentations, laboratory values at admission, treatment regimens, and outcomes were classified according to the categorization. Categorical variables were summarized with numbers and percentages. Quantitative variables were summarized with means and standard deviations or medians and interquartile ranges where appropriate. While chi-square and Fisher's exact test were performed for qualitative variables, the Mann–Whitney U test was used for quantitative variables with the nonparametric distribution.

Univariate, multivariate logistic regression models were used to determine patient characteristics and COVID-19 complications with death. The variables associated with mortality with a *p*-value less than 0.05 were entered into this model. We tried a multivariate risk model using only six vital predictions from univariate models due to the limited sample size. Although acute respiratory distress syndrome and immunosuppression withdrawal were strong predictors of death, they were excluded due to collinearity with cytokine storm. Hazard ratios and the corresponding 95% confidence intervals refer to the increase per unit in a continuous variable. A *p*-value of less than 0.05 is considered significant**.**

## Results

### Patient characteristics

The median age of four hundred and seventy-five participants was 47 years (interquartile range [IQR], 37–56), and 290 (61.1%) of the participants were men. Median follow-up time after COVID-19 was 132 days (IQR, 104–213). One hundred and ninety-five patients (41.1%) were hospitalized, sixty-nine (14.5%) were admitted to the ICU during follow-up, and fifty-three (11.2%) patients required invasive mechanical ventilation. Three hundred and eighty-one patients (89%) in the survivor group and forty-four patients (93.6%) in the non-survivor group were diagnosed with COVID-19 with positive PCR testing (*p* = 0.606). The prevalence of heart disease (*n* = 55 [12.9%] vs. *n* = 15 [31.9%]; *p* < 0.001) and post-transplant diabetes mellitus (*n* = 72 [16.9%] vs. *n* = 17 [36.2%]; *p* = 0.002) was higher in the non-survivor group than in the survivor group. 395 (83.2%) of the patient population underwent kidney transplantation from a living donor.

While there was no significant difference between the groups in terms of anti-T-lymphocyte globulin use (*n* = 240 [56.1%] vs. *n* = 32 [68.1%]; *p* = 0.545), all the patients who received basiliximab induction were survived (*n* = 32[7.5%] vs. *n* = 0; *p* < 0.001). The most common maintenance immunosuppressive agents were tacrolimus, mycophenolate derivatives, and steroids. Table 1 shows the demographic characteristics and immunosuppression regimen of the patients.

**Table 1:** Patients’ demographic characteristics and immunosuppression regimen

	**All patients (*n* = 475)**	**Survivors (*n* = 428)**	**Non-survivors (*n* = 47)**	***p*-value**
**Age (**year)	47 (37–56)	46 (36–55)	54 (44–62)	** < 0.001**
**Sex**				0.095
*Male*	290 (61.1)	256 (59.8)	34 (72.3)	
*Female*	185 (38.9)	172 (40.2)	13 (27.7)	
**Post-transplant follow-up** (months)	76 (41–113)	80 (41–135)	64 (44–100)	0.304
**Etiology of CKD**				
*Hypertensive nephropathy*	49 (10.3)	44 (10.3)	5 (10.6)	0.55
*Diabetic nephropathy*	49 (10.3)	38 (8.9)	11 (23.4)	**0.005**
*Chronic glomerulonephritis*	72 (15.2)	67 (15.7)	5 (10.6)	0.48
*CAKUT*	76 (16)	73 (17.1)	3 (6.4)	**0.037**
*Other*	29 (30.8)	25 (5.8)	4 (8.5)	0.32
*Unknown*	200 (42.1)	181 (42.3)	19 (40.4)	0.92
**Comorbid diseases**				
*Pre-existing lung disease*	33 (6.9)	30 (7)	3 (6.4)	0.583
*Previous heart disease*	70 (14.7)	55 (12.9)	15 (31.9)	**0.001**
*Chronic hypertension*	351 (73.9)	314 (73.4)	37 (78.7)	0.537
*Post-transplant diabetes mellitus*	89 (18.9)	72 (16.9)	17 (36.2)	**0.002**
*Malignancy*	6 (1.2)	5 (1.2)	1 (2.1)	0.897
**Type of donor**				
*Living*	395 (83.2)	356 (83.2)	39 (83)	0.972
*Deceased*	80 (16.8)	72 (16.8)	8 (17)	
**Induction therapy**				
*ATLG*	272 (57.3)	240 (56.1)	32 (68.1)	0.545
*Basiliximab*	32 (6.7)	32 (7.5)	0	** < 0.001**
**Posttransplant complications**				
*Allograft rejection*	53 (11.2)	46 (10.7)	7 (14.9)	0.54
*BK virus nephropathy*	7 (1.5)	7 (1.6)	0	
*CMV viremia*	4 (0.8)	4 (0.9)	0	
**Maintenance immunosuppression at admission**				
Tacrolimus	404 (85.1)	363 (84.8)	41 (87.2)	0.823
Cyclosporine A	38 (8)	37 (8.6)	1(2.1)	0.09
Everolimus	40 (8.4)	35 (8.2)	5 (10.6)	0.36
Sirolimus	6 (12.6)	6 (1.4)	0	0.533
Mycophenolate derivatives	430 (90.5)	388 (90.6)	42 (89.4)	0.466
Azathioprine	19 (4)	18 (4.2)	1 (2.1)	0.421
Steroids	467 (98.3)	422 (98.6)	45 (95.7)	0.182

*Abbreviations*: *CKD* Chronic kidney disease, *CAKUT* Congenital abnormalities of the urinary tract, *RAS* Renin-angiotensin system, *ATLG* Anti-T-lymphocyte globulin, *CMV* Cytomegalovirus

*P*-values ​compared survivors and non-survivors, obtained from the Chi-Square test, Fisher’s exact test, or Mann–Whitney U test. Data were presented as n (%) or median [Interquartile range 25–75] unless otherwise noted

Bold indicates statistically significant associations (*p* < 0.05)

### Clinical presentations

Cough (*n* = 276 [58.1%]), fever (*n* = 249 [52.4%]), and dyspnea (*n* = 162 [34.1%]) were the frequent symptoms at admission, shown in Table 2. Diarrhea (*n* = 92 [21.5%] vs. *n* = 3 [6.4%]; *p* < 0.006) was more common in the survivor group than in the non-survivor group. The oxygen saturation at presentation was lower in deceased patients compared to the surviving patients (98 [IQR, 94–99] vs. 94 [IQR, 88–99]; *p* = 0.006). Lymphocyte count was lower in the deceased patients compared to the survivor patients (1100 [IQR, 700–1650] vs. 743 [IQR, 500–1153]; *p* = 0.033). Although all serum acute phase reactants were higher in deceased patients compared to the surviving patients (Table 2), a statistically significant difference was found in the serum C-reactive protein levels (18 [IQR, 5–49] vs. 66 [IQR, 13–150]; *p* = 0.001), serum ferritin levels (296 [IQR, 113–726] vs. 630 [IQR, 239–1523]; *p* = 0.011), serum interleukin-6 levels (0.23 [IQR, 0.15–1.33] vs. 3.15 [IQR, 1–1.9]; *p* = 0.016), and serum procalcitonin levels (0.09 [IQR, 0.04–3.13] vs. 0.78 [IQR, 0.18–4.15]; *p* < 0.001). The median serum creatinine levels at admission were similar between the survivor and non-survivor groups (1.15 [IQR, 0.9–1.6] vs. 1.2 [IQR, 0.9–1.7]; *p* = 0.762).

**Table 2:** Patients’ clinical characteristics and laboratory results at admission

	**All patients (*n* = 475)**	**Survivors (*n* = 428)**	**Non-survivors (*n* = 47)**	***p*-value**
**Presentation symptoms**				
*Fever*	249 (52.4)	217 (50.7)	32 (68.1)	**0.03**
*Cough*	276 (58.1)	240 (56.1)	36(76.6)	**0.01**
*Dyspnea*	162 (34.1)	129 (30.1)	33 (70.2)	** < 0.001**
*Diarrhea*	95 (20)	92 (21.5)	3 (6.4)	**0.006**
*Anosmia*	23 (4.8)	23 (5.4)	0	0.08
**Initial examination findings**				
*Pulse rate* (/min)	80 (67–89)	80 (65–88)	88 (80–97)	**0.007**
*SpO*_*2*_* value* (%)	98 (94–99)	98 (94–99)	94 (88–99)	**0.006**
*Respiratory rate* (/min)	19 (18–20)	18 (18–20)	22 (18–26)	**0.015**
*Blood pressure* (mmHg)				
Systolic	120 (110–130)	120 (110–130)	120 (120–130)	0.871
Diastolic	80 (70–80)	80 (70–80)	80 (70–80)	1
**Laboratory results at admission**				
Serum creatinine (mg/dL)	1.15 (0.9–1.6)	1.15 (0.9–1.6)	1.2 (0.9–1.7)	0.762
Leucocyte count (/mm^3^)	6300 (4565–8300)	6300 (4500–8300)	6300 (4725–8300)	0.749
Lymphocyte count (/mm^3^)	1000 (670–1595)	1100 (700–1650)	743 (500–1153)	**0.033**
Hemoglobin (g/dL)	12.8 (11.2–15)	12.8 (11.2–14)	12.7 (10.9–14.1)	0.988
Platelet count (/mm^3^)	201 (158–265)	201 (162–266)	183 (131–262)	0.156
Serum CRP levels (mg/L)	20 (5–57)	18 (5–49)	66 (13–150)	**0.001**
Serum ALT levels (IU/L)	20 (14–29)	20 (14–29)	20 (16–36)	0.597
Serum AST levels (IU/L)	20 (16–29)	20 (16–28)	26 (17–35)	**0.032**
Serum LDH levels (IU/L)	238 (189–317)	227 (186–297)	344 (250–537)	** < 0.001**
Serum D-dimer (ng/mL)	386 (201–795)	373 (200–764)	480 (211–1290)	0.54
Serum Ferritin (ng/mL)	354 (130–768)	296 (113–726)	630 (239–1523)	**0.011**
Serum Albumin (g/dL)	4 (3.5–4.3)	4 (3.6–4.3)	3.5 (3.1–4.1)	** < 0.001**
Serum IL-6 (pg/mL)	0.43 (0.15–1.41)	0.23 (0.15–1.33)	3.15 (1–1.9)	**0.016**
Procalcitonin (ng/mL)	0.1 (0.05–0.6)	0.09 (0.04–3.13)	0.78 (0.18–4.15)	** < 0.001**

*Abbreviations*: *SpO2* blood oxygen saturation levels, *CRP* C-reactive protein, *ALT* Alanine aminotransferase, *AST* Aspartate aminotransferase, *LDH* Lactate dehydrogenase, *IL-6* Interleukin 6

*P*-values compared survivors and non-survivors, obtained from the Chi-Square test, Fisher’s exact test, or Mann–Whitney U test. Data were presented as n (%) or median [Interquartile range 25–75] unless otherwise noted

Bold indicates statistically significant associations (*p* < 0.05)

### Treatment regimens

Antimetabolites were discontinued in 428 (90.1%), and both antimetabolites and calcineurin inhibitors were stopped in 49 (10.3%) of the patients. Also, favipiravir was used in 45 (95.7%) non-survivors and 346 (80.8%) survivor patients (*p* = 0.005). Steroid (*n* = 16 [3.7%] vs. *n* = 6 [12.8%]; *p* = 0.015), anakinra (*n* = 14 [3.3%] vs. *n* = 4 [8.5%]; *p* = 0.09), convalescent plasma (*n* = 14 [3.3%] vs. *n* = 7 [14.9%]; *p* < 0.001) were used for the treatment of cytokine storm. While 344 (72.4%) of the patients received anticoagulant treatment, 248 (52.2%) received antibiotics. Duration of hospitalization was longer in deceased patients compared to the survivor patients (9 [IQR, 6–13] vs. 15 [IQR, 8–27]; *p* < 0.001).

### Outcomes

Forty-seven patients (9.9%) of the participants died after a median hospitalization of 15 days (IQR, 8–27). Forty-six of the forty-seven patients (97.9%) who were admitted to the ICU, and received mechanical ventilation, are shown in Table 3. A patient refused treatment in hospital and died at home for an unknown reason two days later. Thirty-five of the patients died due to respiratory failure, ten patients due to multiorgan failure associated with cytokine storm, and one patient due to myocardial infarction. No adverse events occurred during follow-up, and no drug interactions were detected between drugs used to treat COVID-19 and calcineurin inhibitors. One in seven patients in the first six months after transplantation and two out of fifteen in the first year died due to COVID-19.

**Table 3:** Patients’ treatment regimens and outcomes

	**All patients (*n* = 475)**	**Survivors (*n* = 428)**	**Non-survivors (*n* = 47)**	***p*-value**
**Duration of hospitalization** (days)	10 (6–14)	9 (6–13)	15 (8–27)	** < 0.001**
**Withdrawal of IS agent**				
Calcineurin inhibitors	49 (10.3)	32 (7.5)	17 (36.2)	** < 0.001**
Antimetabolites	428 (90.1)	386 (90.2)	42 (89.3)	
**Treatment of infection**				
Hydroxychloroquine	105 (22.1)	95 (22.2)	10 (21.3)	0.89
Favipiravir	391 (82.3)	346 (80.8)	45 (95.7)	**0.005**
Oseltamivir	26 (5.5)	24 (5.6)	2 (4.3)	0.514
**Cytokine‐targeted therapy**				
Anakinra	18 (3.8)	14 (3.3)	4 (8.5)	0.09
Tocilizumab	14 (3.4)	11 (2.6)	3 (6.4)	0.152
Convalescent plasma	21 (4.4)	14 (3.3)	7 (14.9)	** < 0.001**
IVIG	17 (3.6)	12 (2.8)	5 (11.3)	**0.02**
**Anticoagulation**	344 (72.4)	303 (70.8)	41 (87.2)	**0.026**
**Antibiotics**	248 (52.2)	216 (50.5)	32 (68.1)	**0.032**
**Ventilation devices**				
Nasal cannula	158 (33.2)	158 (36.9)	0	** < 0.001**
Non-invasive ventilation	25 (5.3)	24 (5.6)	1 (2.1)	** < 0.001**
Mechanical ventilation	53 (11.2)	7 (1.6)	46 (97.9)	** < 0.001**
**Acute kidney injury**	168 (35.3)	130 (30.4)	38 (80.9)	** < 0.001**
Stage 1	87 (18.3)	73 (17.1)	14 (29.8)	**0.032**
Stage 2	35 (7.4)	26 (6.1)	9 (19.2)	**0.003**
Stage 3	46 (9.7)	31 (7.2)	15 (31.9)	** < 0.001**
**Cytokine storm**	56 (11.8)	30 (7)	26 (55.3)	** < 0.001**
**ARDS**	80 (16.8)	34 (7.9)	46 (97.9)	** < 0.001**
**Hospitalization**	195 (41.1)	195 (45.6)	0	** < 0.001**
**ICU admission**	69 (14.5)	23 (5.4)	46 (97.9)	** < 0.001**

*Abbreviations*; *IS i*mmunosuppression, *IVIG* Intravenous immunoglobulin, *ARDS* acute respiratory distress syndrome, *ICU* Intensive care unit

*P*-values compared survivors and non-survivors, obtained from the Chi-Square test, Fisher’s exact test, or Mann–Whitney U test. Data were presented as n (%) or median [Interquartile range 25–75]

Bold indicates statistically significant associations (*p* < 0.05)

One hundred and sixty-eight (35.3%) patients developed acute kidney injury. There was no difference between the groups in terms of the development of stage one acute kidney injury (*n* = 73 [17.1%] vs. *n* = 14 [29.8%]; *p* = 0.032). However, stage two (*n* = 26 [6.1%] vs. *n* = 9 [19.2%]; *p* = 0.003) and stage three acute kidney injury (*n* = 31 [7.2%] vs. *n* = 15 [31.9%]; *p* < 0.001) were more common in deceased patients than in the survivors. Eight patients (1.9%) in the survivor group and eleven patients (23.4%) in the non-survivor group received dialysis treatment during the COVID-19 follow-up (*p* < 0.001). The occurrence of cytokine storm (*n* = 30 [7%] vs. *n* = 26 [55.3%]; *p* < 0.001) and acute respiratory distress syndrome (*n* = 34 [7.9%] vs. *n* = 46 [97.9%]; *p* < 0.001) was higher in the non-survivor group compared to the survivor group.

**Fig. 4:**
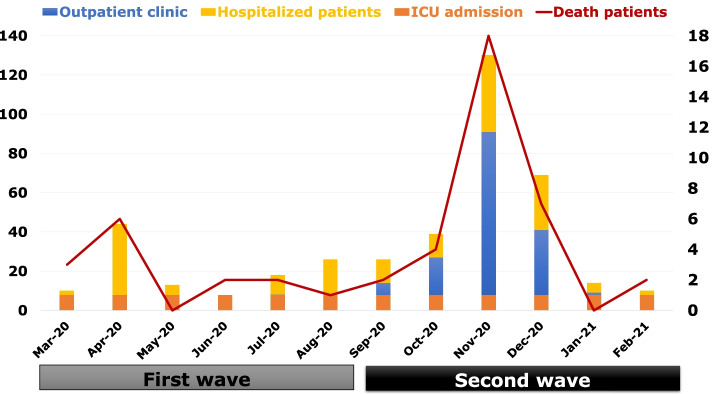
Number of patients according to treatment modalities month by month. Abbreviations: ICU; Intensive care unit

**Fig. 5:**
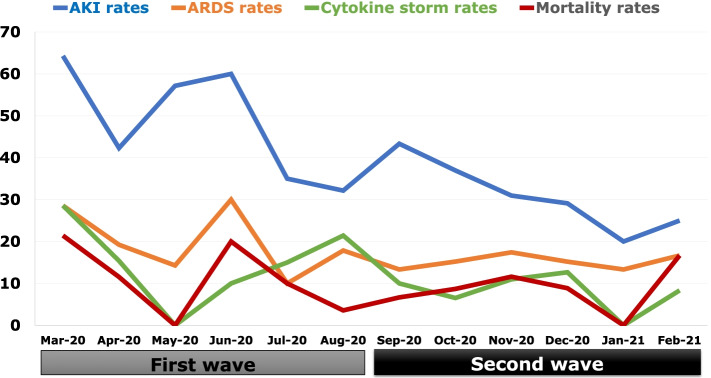
Number of patients according to complications month by month. Abbreviations: AKI; acute kidney injury, ARDS; acute respiratory distress syndrome

The mortality rates were 10.1% and 9.8% in the first and second waves, respectively (Fig. 4 and 5). Hospitalization (*n* = 95 [68.8%] vs. *n* = 100 [29.7%]; *p* < 0.001), acute kidney injury (*n* = 61 [44.2%] vs. *n* = 107 [31.8%]; *p* = 0.009), acute respiratory distress syndrome (*n* = 26 [18.8%] vs. *n* = 54 [16%]; *p* = 0.456), and cytokine storm rate (*n* = 22 [15.9%] vs. *n* = 34 [10.1%]; *p* = 0.072) were higher in the first wave compared to the second wave shown in Table 4.

**Table 4:** Demographic characteristics, laboratory results, and outcomes of the patient’s treatment according to the first two waves

	**All patients (*n* = 475)**	**First wave (*n* = 138)**	**Second wave (*n* = 337)**	***p*-value**
Age	47 (37–56)	46 (36–55)	47 (37–56)	0.456
CRP levels	20 (5–57)	38 (13–78)	14 (4–46)	** < 0.0001**
Lymphocyte	1000 (670–1595)	900 (599–1370)	1100 (700–1700)	**0.012**
LDH levels	238 (189–317)	244 (184–313)	235 (189–319)	0.92
Ferritin levels	354 (130–768)	313 (169–680)	377 (112–806)	0.87
Acute kidney injury	168 (35.4)	61 (44.2)	107 (31.8)	**0.009**
ARDS	80 (16.8)	26 (18.8)	54 (16)	0.456
Cytokine storm	56 (11.8)	22 (15.9)	34 (10.1)	0.072
Outpatient clinic	211 (44.4)	23 (16.7)	188 (55.8)	** < 0.0001**
Hospitalization	195 (41.1)	95 (68.8)	100 (29.7)	** < 0.0001**
ICU admission	69 (14.5)	20 (14.5)	49 (14.5)	0.989
Death	47 (9.9)	14 (10.1)	33 (9.8)	0.907

*Abbreviations: ARDS* Acute respiratory distress syndrome, *ICU* Intensive care unit

*P*-values compared first and second wave obtained from the Chi-Square test or Mann–Whitney U test

Data were presented as n (%) or median [Interquartile range 25–75]

Bold indicates statistically significant (*p* < 0.05)

The 428 surviving patients were followed for a median of 137 (IQR, 113–221) days from the diagnosis of COVID-19. No patient died after the first month of COVID-19 diagnosis. Six (1.4%) of the surviving patients lost allografts during treatment for COVID-19. All were in the pre-dialysis period (eGFR 15–30 mL/min), and immunosuppression except for steroids was discontinued due to severe COVID-19. Acute rejection was diagnosed in two patients (0.5%) after COVID-19. The first patient was examined for graft dysfunction (eGFR: 16 mL/min) that persisted for forty days after COVID-19, and de novo DSA was detected. Glomerulitis and peritubular capillaritis were detected in the allograft biopsy. Allograft function returned to baseline (eGFR:65 mL/min) with plasmapheresis and IVIG treatment. In the second patient, interstitial inflammation and tubulitis were detected in the kidney biopsy performed due to graft dysfunction (eGFR: 24 mL/min) that persisted for twenty days after COVID-19, and 500 mg methylprednisolone treatment was administered for three days. The graft function improved (eGFR:74 mL/min) and returned to baseline.

One hundred thirty-eight and three hundred sixty-nine patients were followed for more than six and three months, respectively.

There was no difference in the median serum creatinine clearance of the surviving patients at baseline (52 mL/min [IQR, 47–66]), first- (56 mL/min [IQR, 51–68]), third- (51 mL/min [IQR,48–67]) and sixth-months (52 mL/min [IQR, 48–81]).

### Predictors of mortality

Patients with posttransplant diabetes mellitus were associated with a twofold increased risk of death (2.876 [95% CI, 1.341–6.17; *P* = 0.004]). Multivariate logistic regression analysis revealed that the development of cytokine storm (17.855 [95% CI, 8.625–17.826; *P* < 0.001]) was an independent predictor for mortality, shown in Table 5.

**Table 5:** Logistic regression analysis of mortality risk factors for kidney transplant recipients

	**Univariate analysis**			**Multivariate analysis**		
	**Odds ratio**	**Confidence interval**	***p*-value**	**Odds ratio**	**Confidence interval**	***p*-value**
**Age > 65 (vs < 65)**	**1.048**	**1.022–1.075**	** < 0.001**	1.833	0.562–5.977	0.315
**ATLG induction**	1.549	0.788–3.045	0.205			
**Previous heart disease**	**3.162**	**1.609–6.213**	**0.001**	1.719	0.665–4.442	0.263
**PTDM**	**2.982**	**1.544–5.758**	**0.001**	**2.876**	**1.341–6.17**	**0.007**
**Development of AKI**	**3.782**	**2.036–7.028**	** < 0.001**	1.058	0.462–2.424	0.894
**Cytokine storm**	**16.425**	**8.285–32.564**	** < 0.001**	**17.855**	**8.625–17.826**	** < 0.001**

*Abbreviations*: *AKI* Acute kidney injury, *ATLG* Anti-T-lymphocyte globulin, *PTDM* Posttransplant diabetes mellitus

Bold indicates statistically significant (*p* < 0.05)

## Discussion

COVID-19 is a health disaster, and kidney transplant recipients are at high risk of severe illness due to COVID-19. This study investigated 475 patients with a median follow-up of 132 days after COVID-19. One hundred and ninety-five patients were hospitalized, and sixty-nine were admitted to the ICU. Forty-seven patients died after a median length of hospitalization of 15 days. One hundred and sixty-eight patients developed acute kidney injury. Cytokine storm occurred in fifty-six patients, and acute respiratory distress syndrome developed in eighty patients. The development of cytokine storm was the risk factor for mortality. The 428 surviving patients were followed for a median of 137 days from the diagnosis of COVID-19. No patient died after the first month of COVID-19. Six of the surviving patients lost allografts during treatment for COVID-19.

In European centers, the mortality rates of 32% in kidney transplant recipients have been reported in the early wave of the pandemic, then; mortality rates have been decreased to 25% with the standardization of treatment regimens, improvement of medical practices, early diagnosis, and treatment [13–15]. However, the mortality rate in most US centers is over 30% [16]. In our study, the mortality rate was 10.1% in the first and 9.8% in the second wave. This difference in mortality rate may explain by several reasons. First, the median age in our study was 47, in the European Registry 60, and in the international TANGO consortium 62. Second, the prevalence of diabetes mellitus in our study was 18.9%, the European registry of 32%, and the TANGO consortium of 52.1%. Third, 83.2% of our patients received allografts from living donors, while only 22.7% of the patients in the TANGO consortium had living donors [14, 16]. Also, in a study from India, the mortality rate was 11.6%, similar to ours. In this study, the mean age was 43 years, the frequency of diabetes was 32%, and 90.4% of the patients received a kidney from a living donor [15]. On the other hand, there is no one-to-one comparison between low and high mortality patients; and further research exploring this topic may be interesting. In addition, a recent article showed the negative impact of the short time between transplantation and COVID-19 on clinical prognosis (19). Our study showed a limited increase in the mortality rate in patients with COVID-19 in the early post-transplant period, but causality analysis was not done due to the small number of patients.

Acute kidney injury and graft loss are common in kidney transplant recipients with COVID-19. The direct toxicity of the virus, tubular damage from cytokine storm, reduction in circulating volume, multiple organ failure, and reduced immunosuppression during disease are the possible mechanisms [6, 13, 15–17]. Risk factors include permanent graft damage before COVID-19, severe COVID-19, and stage 3 acute kidney injury during COVID-19 [18, 19]. While the incidence of acute kidney injury was between 42 and 52%, graft loss ranged between 8–10% in recent articles. In addition, dialysis-induced acute kidney injury was associated with high mortality [19]. In our study, 168 (35.3%) of our patients had acute kidney injury during COVID-19, 19 patients needed dialysis, and 11 of those who received dialysis died. In addition, six (1.4%) of the surviving patients lost their grafts. The lower incidence of acute kidney injury and graft loss compared to recent articles may be explained by the that most of the patients were living kidney transplant recipients with preserved kidney function, mild COVID-19, and a low incidence of stage III acute kidney injury.

Cytokine storm was a powerful predictor of death in COVID-19. Also, higher levels of interleukin-6 are associated with shorter survival. Cytokine storm treatment in COVID-19 is an unsolved problem because cytokine-targeted therapy may impair clearance of SARS-CoV-2, increase the risk of secondary infections, and cause worse outcomes [16, 17, 20–23]. Tocilizumab was used for cytokine storm treatment in many prospective randomized studies. However, conflicting results were obtained in these studies, and there is no consensus yet [24–26]. Steroid, intravenous immunoglobulin, convalescent plasma, anakinra, and tocilizumab were used for the cytokine storm treatment in our study. However, we think that this study is not appropriate to evaluate the efficacy of these drugs due to changes in treatment algorithms during the pandemic. More standardized, double-blind, prospective, randomized controlled studies are needed to investigate the effect of cytokine-targeted therapy in COVID-19 patient with cytokine storm. Also, there is an ongoing nomenclature debate on the definition of immune response to COVID-19; we used the term cytokine storm, similar to others cited in our study [23, 26].

Many differences have been described between the first and second waves of the pandemic, with a higher proportion of younger cases, milder presentation, and lower mortality. These differences are evident in Spain (25.8% in the first, 16.7% in the second wave), where patient care is standardized and improved, but a significant difference is not observed in cases where the hospitals are overcrowding, and therefore patient care cannot be improved, such as in India [27–29]. The decrease in acute kidney injury and hospitalization rates was determined in the second wave of the pandemic compared to the first wave in our study, but this improvement was limited compared to the Spanish Study. Several reasons may explain this limitation. First, the new daily cases in the second wave were higher than in the first wave in our study, which differs from the Spanish Registry. Second, the patients in the second wave were older than in the first wave in our study contrast to the Spanish report [30]. On the other hand, the death rate in both waves of the Spanish trial was higher than ours. Interestingly, kidney transplant recipients with COVID-19 in India have a higher incidence of mucormycosis, unlike in the world [29].

The new antiviral agents are still under investigation in COVID-19. Remdesivir, which is currently widely used in most countries, has reduced the length of stay in hospitalized patients [9, 31]. Also, favipiravir was an RNA polymerase inhibitor used as an antiviral treatment for COVID-19. Major side effects were hyperuricemia and abnormal liver function. Favipiravir did not interact with calcineurin inhibitors due to the non-cytochrome metabolic pathway of favipiravir [32]. Favipiravir was the most commonly used antiviral agent in our study. Any significant toxicity and drug interactions were not observed associated with favipiravir. In addition, our study is not appropriate for investigating the efficacy of favipiravir in kidney transplant recipients. Also, the RECOVERY trial showed that dexamethasone decreased mortality in patients with COVID-19 on respiratory support. The effect is prominent in those who do not receive dialysis treatment [12]. In our study, the steroid dosage was increased in patients who needed ventilator support.

Our study has several limitations; the follow-up period was short, it did not include the control group or pediatric cases, and PCR samples were collected from symptomatic patients rather than all patients. Also, our findings were obtained from unvaccinated kidney transplant recipients; therefore, our results may differ from infections with new SARS-CoV-2 variants and unvaccinated or partially vaccinated patients. In addition, due to the high false-negative results of RT-PCR in the first three months of the pandemic, some patients were diagnosed with COVID-19 by computed tomography alone. Also, data on the tracking of SARS-CoV-2 variants, antibody measurements, and long-term symptoms of COVID were not collected. These limitations suggested that we could not draw firm conclusions from these experiences; therefore, our findings should be validated by prospective cohort studies. Using a standard immunosuppressive treatment regimen can be a strength of the study.

In conclusion, this study shows that cytokine storm was a risk factor for death. The deaths occurred in the first month of COVID-19. Also, acute kidney injury is common in kidney transplant recipients with COVID-19, and some of them suffer from allograft rejection and graft loss after the initial acute episode.

## Data Availability

The data that support the findings of this study are available from the corresponding author upon reasonable request.

## Abbreviations

AKI: Acute kidney injury
ALT: Alanine aminotransferase
ARDS: Acute respiratory distress syndrome
AST: Aspartate aminotransferase
ATLG: Anti-T-lymphocyte globulin
CAKUT: Congenital abnormalities of the urinary tract
CKD: Chronic kidney disease
COVID‐19: Coronavirus disease 2019
CMV: Cytomegalovirus
CRP: C-reactive protein
eGFR: Estimated Glomerular Filtration Rate
FiO2: The inspiratory oxygen fraction
IS: Immunosuppression
IVIG: Intravenous immunoglobulin
IL-6: Interleukin-6
IQR: Interquartile range
ICU: Intensive care unit
LDH: Lactate dehydrogenase
PaO2: Partial pressure of arterial oxygen
PTDM: Posttransplant diabetes mellitus
RAS: Renin-angiotensin system
RT-PCR: Real-time polymerase chain reaction
SARS-CoV-2: Severe acute respiratory syndrome coronavirus 2
SpO2: Blood oxygen saturation levels
